# Section 17 Leave Utilisation and Outcomes in Acute Adult Psychiatric Inpatients: A Closed-Loop Audit

**DOI:** 10.1192/bjo.2025.10687

**Published:** 2025-06-20

**Authors:** Tang Song Ling, Melanie Noad, Victor Asamoah

**Affiliations:** 1 Hertfordshire Partnership University NHS Foundation Trust, Hertfordshire, United Kingdom; 2 East and North Hertfordshire NHS Trust, Hertfordshire, United Kingdom

## Abstract

**Aims:** Evaluate the utilisation frequency and outcomes of Section 17 leave in an acute adult inpatient psychiatric unit.

Improve the quality of care through evidence-based and individualised treatment plans.

Inform ward resource allocation related to Section 17 leave.

**Methods:** Data collection: The audit included all seven patients admitted to New Victoria Court from April to May 2024. Data were collected from electronic patient records, documented discussions with patients and carers, and direct interviews with service users to capture experiences, benefits, and challenges of leave. Nursing colleagues were also interviewed about the long-term feasibility of this initiative.

Standard: HPFT Section 17 Leave of Absence Policy.

Intervention: A leave feedback template was designed and implemented to record leave outcomes daily, completed by the safety nurse at the end of each shift.

Data analysis: Quantitative measures included the percentage of compliance with documentation standards, incidents, and what went well during leave. Qualitative data enriched the understanding of leave’s impact on recovery and ward staff capacity.

A re-audit was performed two weeks post-intervention using similar parameters.

**Results:** Quantitative findings: The total number of leave episodes decreased from 157 to 137, likely due to one fewer patient on the ward post-intervention.

There were no significant changes in the proportion of ground or community leave utilised.

Notably, the percentage of documented leave outcomes increased by 13.2%, and documentation of what went well during leave rose by 50.3%.

Incidents during leave decreased from 8.8% to 0%, though patient demographics and mental state changes might have confounded this.

Qualitative findings: Patient feedback revealed mixed experiences. Some patients valued leave for accessing the community, viewing it as beneficial for recovery. Others expressed frustration with restrictions, preferring discharge over limited leaves. One patient reported no need for leave at all.

Nursing colleagues supported documenting leave outcomes but highlighted concerns about additional workload. Some feedback forms were used to record general observations rather than leave-specific outcomes, requiring clarification during data analysis.

**Conclusion:** This audit demonstrated a significant improvement in the documentation of leave outcomes, supporting evidence-based and individualised patient care. Stable utilisation of ground and community leave aids ward resource allocation. The reduced incidents might reflect improved monitoring and risk management.

Action plan:

Implement a standardised leave feedback template for regular review during ward rounds.

Conduct training for staff on thorough documentation and utilising the template.

Ongoing discussion between the trust audit team and medical directors regarding trust-wide implementation of this initiative.

